# The impact of dietary supplements on blood pressure in older adults: A network meta-analysis of randomized controlled trials

**DOI:** 10.1016/j.heliyon.2024.e25615

**Published:** 2024-02-08

**Authors:** Agnieszka Kujawska, Sabri Bromage, Jose Augusto Simoes, Jūratė Zupkauskienė, Nicholas McMahon, Paweł Zalewski, Sławomir Kujawski

**Affiliations:** a Department of Exercise Physiology and Functional Anatomy, Ludwik Rydygier Collegium Medicum in Bydgoszcz Nicolaus Copernicus University in Toruń, Świętojańska 20, 85-077, Bydgoszcz, Poland; b Institute of Nutrition, Mahidol University, Salaya, Thailand; c Department of Nutrition, Harvard T.H. Chan School of Public Health, Boston, USA; d Department of Medical Sciences, University of Beira Interior, 6200-506, Covilha, Portugal; e Clinic of Cardiac and Vascular Diseases, Vilnius University, 08661, Vilnius, Lithuania; f School of Human Movement and Nutrition Sciences, University of Queensland, St. Lucia, QLD, Australia; g Department of Experimental and Clinical Physiology, Laboratory of Centre for Preclinical Research, Warsaw Medical University, 1b Banacha Street, 02-097, Warsaw, Poland

**Keywords:** Hypertension, Older adults, Dietary supplements, Nutritional supplementation, Elderly

## Abstract

**Purpose:**

The prevalence of hypertension (HTN) increases with age and there is a need for effective, evidence-based treatments for HTN among older adults. The objective of this study was to perform a network meta-analysis to evaluate the effectiveness of different forms of nutritional supplementation on reducing blood pressure in older adults.

**Methods:**

A systematic review using PubMed and Clinical Key was performed to identify randomized controlled trials (RCTs) evaluating the effects of dietary supplements on blood pressure in adults older than 65 years of age. Network meta-analysis (NMA) was used to compare and rank the effects of different supplements on systolic (sBP), diastolic (dBP), and mean (mBP) blood pressure. Supplements were ranked according to P score. Meta-regressions were conducted to examine whether treatment effects were moderated by baseline BP and supplementation duration.

**Findings:**

We identified 144 relevant studies in the literature, twelve of which met criteria for inclusion in NMA. The included studies were published between 2003 and 2022. In reducing sBP, docosahexaenoic acid (DHA) and eicosapentaenoic acid (EPA), inorganic nitrates, tart cherry juice, and vitamin D supplementation were more effective than placebo, and the effect of tart cherry juice outranked that of vitamin D, vitamin E, and vitamin K2. In reducing dBP, inorganic nitrates, DHA and EPA, protein, resveratrol, and vitamin D supplementation were more effective than placebo, and the effect of resveratrol outranked that of tart cherry juice, vitamin D, vitamin E, and vitamin K2. However, the effects of tart cherry juice on sBP and resveratrol on dPB were smaller than the pooled effect of placebo, and none of the pairwise differences between the effects of examined supplements were statistically significant. Caution is needed when interpreting these results given concerns about the risk of bias assessed in seven of the twelve studies included in this analysis.

## 1 Introduction

The prevalence of hypertension (HTN) is two times higher in older adults than in younger populations [1]. The exact mechanism underlying hypertension development is not yet fully understood, however, there are multiple factors that contribute to its development [2]. Physiological aging might be related to multisystemic changes, which are related to HTN development. Aging can lead to a decrease in the quality of blood vessels (chronic inflammation, endothelial dysfunction, a decrease in elastin, an increase in collagen, and calcification of arteries) and an increase in a number of factors that contribute to control over blood pressure, namely in sympathetic nervous system activity, and aldosterone production and salt sensitivity [3]. In turn, these factors might be related to HTN development.

The American College of Physicians and the American Academy of Family Physicians recommend that antihypertensive medications be used to lower systolic blood pressure (sBP) when sBP is chronically at or higher than 150 mm Hg to reduce the risk of stroke, cardiac events, and mortality [4]. HTN treatment options include angiotensin-converting enzyme inhibitors, thiazide-like diuretics; angiotensin receptor blockers, calcium-channel blockers, and beta-blockers [5].

The effects of different forms of dietary supplementations on reducing blood pressure in older adults remain uncertain [6,7]. The most recent American College of Cardiology (ACC) and/or American Heart Association (AHA) and European Society of Cardiology (ESC) and/or European Society of Hypertension (ESH) guidelines advise a diet rich in fruits, vegetables, whole grains, and low-fat dairy products with reduced content of saturated and total fat, weight reduction for overweight patients, reduction of intake of dietary (Na^+^) and increase intake of dietary (K^+^) and moderation in alcohol intake [8]. In home telehealth remote monitoring is discussed [8]. However, despite a number of randomized controlled trials (RCTs) studying the effects of dietary supplements on BP in older adults, there are no clear guidelines on optimal dietary supplementation strategies.

A recent network meta-analysis (NMA) protocol included the assessment of various types of dietary supplements, including vitamins, myo-inositol, choline, minerals, probiotics, prebiotics, synbiotics, and omega-3 fatty acids on blood glucose and lipid metabolism in gestational diabetes mellitus patients [9]. In this NMA of randomized controlled trials (RCTs) we aimed to assess both direct and indirect effectiveness of dietary supplements in reducing resting blood pressure (BP) compared to placebo in older adults (>65 years).

## 2 Methods

The analysis was conducted and reported in accordance with the Preferred Reporting Items for Systematic Reviews and Meta-Analyses (PRISMA) guidelines [10,11].

### 2.1 Search strategy

The PubMed and Clinical Key electronic databases were systematically searched for RCTs that examine the effects of dietary supplements on the reduction in BP in older adults from inception to December 01, 2022. The following search terms were applied: (supplement or supplementation) blood pressure (older people OR older). The search was restricted to clinical trials and papers in English or Polish. The search was supplemented by cross-matching reference lists, key author searches, and citation searching of all retrieved papers to potentially identify additional studies. Annex 1 of the supplemental material details the search strategy. This systematic review was registered at the International Prospective Register of Systematic Reviews (PROSPERO) with the protocol number CRD42021290720.

### 2.2 Eligibility criteria

Selection criteria for all relevant articles were determined by two researchers (AK and SK). The eligibility criteria are detailed below using the participants, intervention, controls, outcomes, and study design (PICOS) framework: (i) Participants: we included studies enrolling participants with mean age ≥65 years in placebo and supplemented groups(s) [12]. This age criterion was chosen based on convention in the literature on ageing [12]. In addition, 2018 ESC/ESH guidelines defined ‘old’ as ≥65 years [13]. Authors of 2018 ESC/ESH guidelines underlined the significance of BP reduction in older patients with baseline SBP ≥160 mmHg to reduce cardiovascular event risk [13]; (ii) Interventions: RCTs assessing dietary supplementation compared with placebo in the form of supplementation programme excluding studies assessing the acute response to single supplement administration only (iii) Controls: groups receiving placebo. Studies without placebo conditions were excluded. (iv) Outcomes: the outcome measures were changes in BP before vs after intervention. Studies not providing central and dispersion values of BP before and/or after intervention or its change were excluded (v) Studies: only RCTs or crossover trials were included in the analysis (Table 1).

**Table 1:** Meta-analysis description according to PICO.

Population Patient Problem	Intervention	Comparison	Outcome
Studies enrolled participants with mean age ≥65 years in placebo and supplemented groups(s).	RCTs assessing dietary supplementation compared with placebo in the form of supplementation programme	Groups receiving placebo	The outcome measures were changes in BP before vs after intervention.

### 2.3 Data extraction

After the removal of duplicates, two researchers (AK and SK) screened the titles and abstracts according to the pre-specified criteria in an independent manner. Full papers of abstracts potentially eligible for inclusion were then screened (AK and SK). In cases of disagreement, a third researcher (JAS) was consulted for a final decision. Data extraction was completed by two researchers (AK and SK) in an independent manner. Information on study characteristics, description of examined sample, interventions, and outcomes were extracted from each study.

### 2.4 Quality assessment

Cochrane's risk of bias tool was used by two researchers (AK and SK) to independently assess the risk of bias, including random sequence generation, allocation concealment, blinding of participants and personnel, blinding of outcome assessment, incomplete outcome data, selective reporting, and other, unspecified by criteria [14]. Each quality assessment and overall assessment was classified as low risk of bias, some concerns, or high risk of bias.

### 2.5 Statistical analysis

Both transitivity (equal distribution of effect modifiers across trials), homogeneity, and consistency of analysed trials were assumed [15]. The I2 statistic and corresponding p-values were calculated as a measure of the statistical heterogeneity, with I2 ≥50% indicating substantial heterogeneity [16]. Network meta-analysis (NMA) was used to assess the effects of different types of dietary supplementation compared to placebo in the reduction of BP. The results are presented as the mean differences (MDs) and 95% confidence intervals (CIs) [17]. The network is presented in a circular ordering of the vertices. Each node (point) represents an intervention, and the size of the point is proportionally related to the square root of the sample size from each group (combined in the case of intervention was conducted on more than two samples). The color of the point denotes the result of the overall risk of bias of studies that incorporated a particular supplement. Green color denotes a low risk of bias, yellow denotes some concerns and red has a high risk of bias. Placebo is denoted by all three colors proportionally to the number of studies with a particular risk of bias that included placebo. Edges (lines) between nodes (points) represent the direct comparison of evidence, and its thickness is related to weight from random effect meta-analysis comparing two treatments. The number of studies which examine the effects of a particular supplement is denoted on the line. Multi-arm studies are denoted with a turquoise color. Supplementation interventions were ranked according to their P score, which is between 0 and 1. The NMA can estimate the best effects of each intervention on different outcomes and rank each nutritional supplementation based on P score values. The larger P score values indicate a better effect of intervention. P scores are based solely on the point estimates and standard errors of the network estimates, and measure the mean extent of certainty that one intervention is better than another, averaged over all competing interventions [18]. The greater probability of the particular supplementation being ranked as best is shown by a higher P score [18]. Supplementation interventions with a score close to 0.5 are likely to be of similar effectiveness because the mean P score is 0.5. A confidence interval of each comparison should be interpreted in conjunction with the P score. League tables were created to show a direct comparison of diet supplements [19]. Step rankograms show cumulative ranking probability and were created using 1000 simulations. Meta-regression was done to assess the correlation between baseline blood pressure values and supplementation duration expressed in weeks with weighted mean differences. A comparison-adjusted funnel plot was used to assess publication biases and small sample effects were examined. All results presented come from random effect meta-analysis. The netmeta package was used to conduct the NMA [20]. Meta-regressions were conducted in R using the rma function in metafor package [21]. All analyses were performed with a significance level α = 0.05.

## 3 Results

### 3.1 Study selection and characteristics

**Fig. 1:**
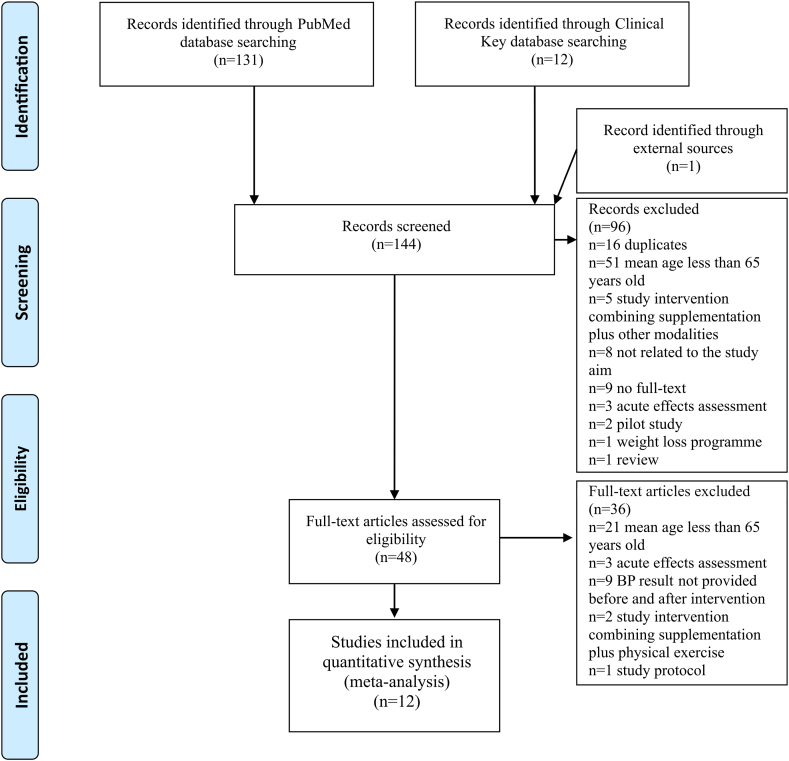
Flow chart of study selection. *A total of 144 articles were retrieved initially from investigated databases. After inspection of the titles and abstracts, 48 studies were selected for further review. Eventually, twelve studies met our inclusion criteria*.

A total of 144 articles were retrieved initially from investigated databases. After inspection of the titles and abstracts, 48 studies were selected for further review. Eventually, twelve studies met our inclusion criteria (Fig. 1). The included studies were published between 2003 and 2022. In overall, 2736 participants were included in this review in the intervention groups and 2631 in placebo. The mean age of the participants was 72.84 in the intervention and 73.16 in the placebo group, respectively. The mean duration of the supplementation was 45.68 weeks (from 4 to 156.43) (Table 2). Samples included both normotensive and hypertensive participants in five studies [[22], [23], [24], [25], [26]], one study included 90% of patients with hypertension in study groups [27], four studies did not report the prevalence of hypertension in the examined groups [[28], [29], [30], [31]] and two studies did not include patients with hypertension [32,33].

**Table 2:** Characteristics of included studies.

First author	Supplementation	Sample age (mean ± SD)	Sample size (n)	Sex (% of females)	Blood pressure status (hypertensive)	Anti-hypertensive medication status	Baseline systolic blood pressure (mean mmHg±SE)	Baseline diastolic blood pressure (mean mmHg±SE)	Intervention duration (weeks)
Aaron C. Schneider et al. (2018) [28]	NO2− 20 mg/d, NO3− 250 mg/d	67 ± 1	13	N/A	N/A	N/A	125 ± 4	78 ± 3	4
	placebo	67 ± 1	13	N/A	N/A	N/A	122 ± 3	74 ± 2	
Joseph Tomson et al. (2017) [22]	vitamin D 4000 IU/d	71 ± 6	102	49%	39%	49%	132.7 ± 21.1	78 ± 11.3	52.1
	vitamin D 2000 IU/d	72 ± 6	102	50%	43%	51%	131.8 ± 17.1	76.6 ± 10.3	
	placebo	72 ± 6	101	49%	35%	46%	129.5 ± 18.8	76.6 ± 12.1	
Jennifer A. McMahon et al. (2007) [23]	folate 1 mg, vitamin B-12 500 mg, vitamin B-6 10 mg/d	73.6 ± 5.8	126	37%	25%	44%	136.6 ± 1.9	74.8 ± 1.2	104.3
	placebo	73.3 ± 5.7	126	52%	30%	40%	138 ± 1.8	75.8 ± 1	
Stephen D. Anton et al. (2014) [24]	resveratrol 300 mg/d	73.17 ± 2.08	12	50%	45%	N/A	125.54 ± 3.2	71.5 ± 2.8	12.9
	resveratrol 1000 mg/d	73.6 ± 2.53	10	50%	20%	N/A	132.06 ± 5.5	73.5 ± 3.7	
	placebo	73.3 ± 2.06	10	50%	70%	N/A	136.1 ± 3.4	77.15 ± 2.9	
Anna E. Stanhewicz et al. (2015) [30]	folic acid 5 mg/d	71 ± 3	11	55%	0%	0%	121 ± 3	74 ± 3	6
	placebo	71 ± 3	11	55%	0%	0%	121 ± 3	74 ± 3	
James V. Jessup et al. (2003) [31]	vitamin E 400 IU/d	76.1 ± 4.3	15	N/A	0%	N/A	140.5 ± 4.2	74.7 ± 2.5	16
	placebo	76.9 ± 4.5	15	N/A	0%	N/A	140.5 ± 5	71.2 ± 2.6	
Heike A. Bischoff-Ferrari et al. (2020) [25]	vitamin D 2000 IU/d	75 ± 4.5	1076	62%	40%	51%	144.2 ± 0.03	76 ± 0.02	156.4
	placebo	74.9 ± 4.4	1081	61%	39%	48%	142.9 ± 0.03	75.7 ± 0.02	
	DHA 660 mg/d + EPA 330 mg/d	74.7 ± 4.3	1073	62%	39%	47%	143.2 ± 0.03	75.8 ± 0.02	
	placebo	75.2 ± 4.6	1084	61%	40%	52%	143.9 ± 0.03	75.9 ± 0.02	
Jonathan M. Hodgson et al. (2012) [26]	protein 30 g/day	74.3 ± 2.7	93	100%	N/A	50%	133.9 ± 0.2	66.7 ± 0.2	104.3
	placebo	74.3 ± 2.6	87	100%	N/A	52%	134.2 ± 0.2	67.4 ± 0.2	
R.L. Fulton et al. (2016) [27]	vitamin K2 100mcg/d	76.0 ± 4.4	40	47%	90%	75%	144 ± 2.7	81 ± 1.7	26.1
	placebo	77.1 ± 4.8±	40	42%	90%	63%	148 ± 3.2	83 ± 1.6	
Sheau C. Chai et al. (2018) [27]	tart cherry juice 480 ml/d	70 ± 3.7	17	60%	N/A	N/A	141.4 ± 6.5	79.7 ± 4.1	12
	placebo	69.5 ± 3.9	17	47%	N/A	N/A	133.4 ± 3.6	78.1 ± 2.3	
Salvador J. Jaime (2022) [30]	l-citrulline 6 g/d	N/A	16	N/A	N/A	N/A	137 ± 3.3	77 ± 2	2
	placebo	N/A	16	N/A	N/A	N/A	138 ± 3.3	77 ± 1.8	
Mengelberg et al. (2021) [31]	DHA 1491 mg/d + EPA 351 mg/d	72.3 ± 6.2	30	53%	N/A	53%	145.9 ± 2.9	83.6 ± 2.2	52.1
	placebo	73.4 ± 7	30	63%	N/A	57%	140.2 ± 4	80.1 ± 2.5	

### 3.2 Results of Risk of Bias

**Fig. 2:**
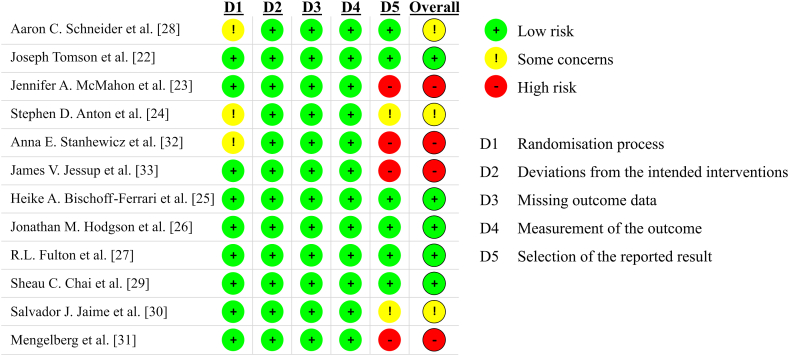
Results of Risk of Bias analysis of single studies. A plus inside a green dot shows low risk, an exclamation mark inside a yellow dot indicates some concerns regarding bias, and a minus inside a red dot shows a high risk of bias.

Bias in the specific studies is shown in Fig. 2. Bias related to randomization was assessed as low in five of the twelve analysed studies (Fig. 2). Randomization of participants was done in all analysed studies, however, in three of these studies, there is a lack of description of specific methods [24,28,32] (Fig. 2) Therefore, some concerns might be related to a potential risk of bias related to randomization. The risk of bias related to the potential selection of the reported results was assessed as high or including some concerns in four studies. Four did not include information if the statistical analysis was conducted according to pre-specified protocol, nor on registration of trial [30,[32], [33], [34]], therefore risk of bias was assessed as high (Fig. 2). Two studies were registered [24,31]. However, in one study [24] changes in blood pressure were not included in the primary or secondary outcomes. Therefore, it was assumed that some concerns might apply to the risk of bias related to the result selection [24] (Fig. 2). Another study was prospectively registered, and changes in blood pressure are denoted as a secondary outcome [31]. Therefore, also some concerns might apply regarding the risk of bias in the included studies (Fig. S1).

### 3.3 Network plots

**Fig. 3:**
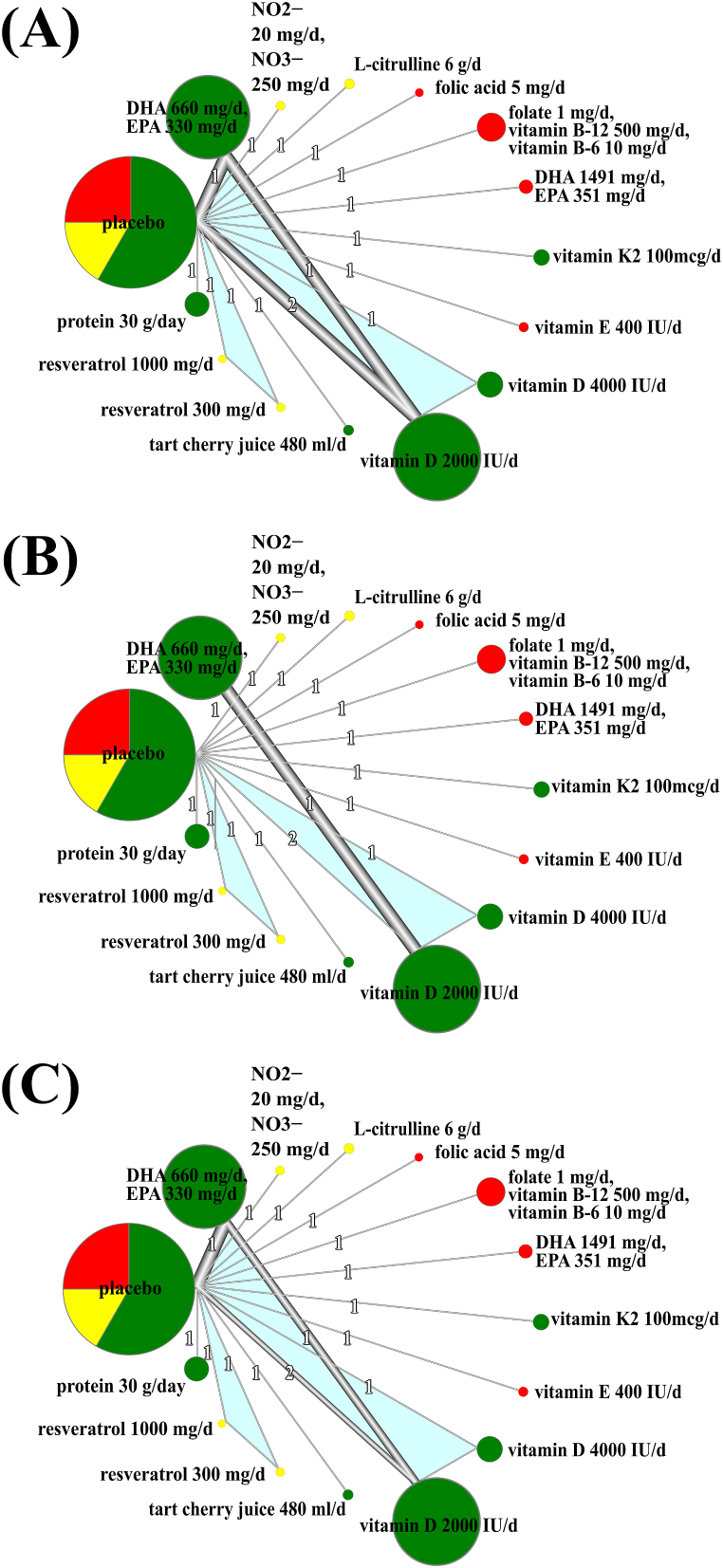
Network analysis of treatment effects on A: systolic blood pressure, B: diastolic blood pressure, C: mean blood pressure. Each node (point) represents an intervention, and the size of the point is proportionally related to the square root of the sample size from each group (combined in the case of intervention was conducted on more than two samples). The color of the point denotes the result of the overall risk of bias of studies that incorporated a particular supplement. Green color denotes low risk of bias, yellow denotes some concerns, and red high risk of bias. Placebo is denoted by all three colors proportionally to a number of studies with a particular risk of bias that included placebo. Multi-arm studies are denoted with a turquoise color. Edges (lines) between nodes (points) represent the direct comparison of evidence, and its thickness is related to weight from random effect meta-analysis comparing two treatments. The number of studies which examine the effects of a particular supplement is denoted on the line.

Fig. 3A, B, and 3C show the network plots of the treatments' effects on sBP, diastolic blood pressure (dBP) and mean blood pressure (mBP), respectively. Fifteen interventions from twelve studies were included in the network meta-analysis: combination of docosahexaenoic acid (DHA) 1491 mg/d and eicosapentaenoic acid (EPA) 351 mg/d, DHA 660 mg/d + EPA 330 mg/d, inorganic nitrate (NO2- 20 mg/d, NO3- 250 mg/d), l-citrulline 6 g/d, folic acid 5 mg/d, combination of folate 1 mg, vitamin B-6 10 mg/d and vitamin B-12 500 mg, vitamin K-2 100mcg/d, vitamin E vitamin E 400 IU/d, vitamin D 4000 IU/day, vitamin D 2000 IU/day, tart cherry juice 480 ml/d, resveratrol 300 mg/day, resveratrol 1000 mg/day, protein 30 g/day, and placebo.

### 3.4 Results of network meta-analysis

For network meta-analyses both for sBP, dBP, mBP tau^2 = 0; tau = 0; I^2 = 0%. The inconsistency test showed that sBP (Q = 0.44,p = 0.5), dBP (Q = 0.10,p = 0.75), and mBP (Q = 0.23,p = 0.63) exhibited no inconsistencies in the global analysis at the levels of alpha value = 0.05, indicating that the between design was consistent across studies.

#### 3.4.1 Pairwise comparison of effects of diet supplements on systolic blood pressure in older adults

**Fig. 4:**
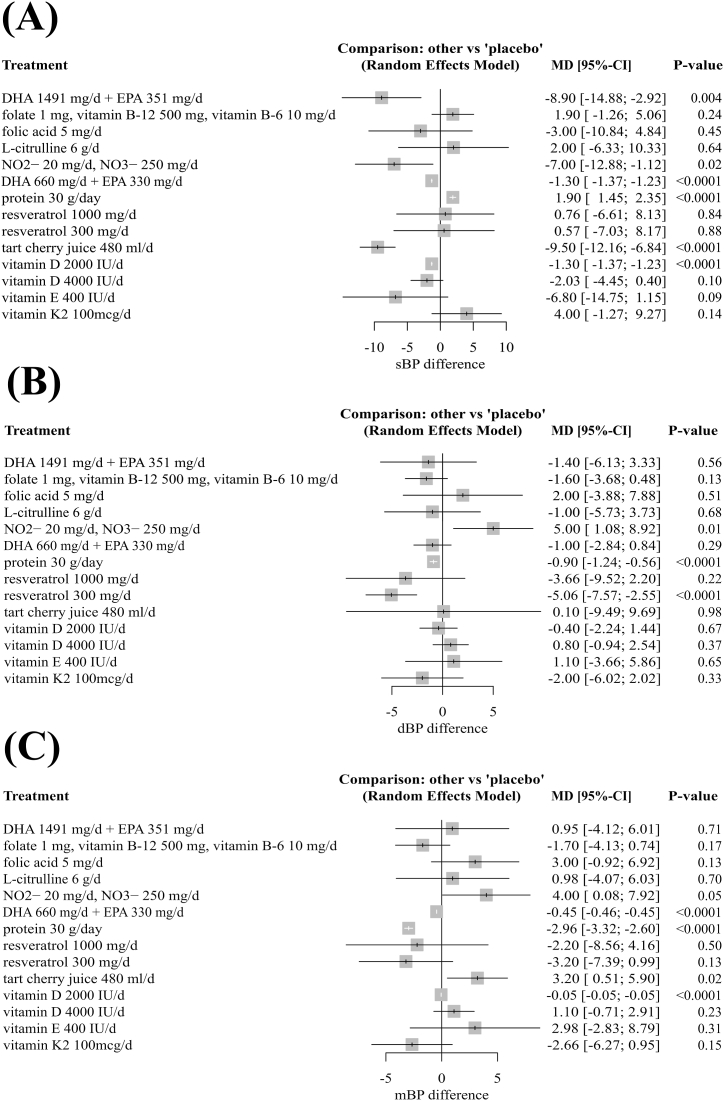
Pairwise comparison to placebo. *Effects of diet supplements on a) systolic blood pressure, b) diastolic blood pressure, c) mean blood pressure*.

Fig. 4A shows the results of the effects of diet supplements on sBP. Compared with placebo, supplementation with DHA 1491 mg/d + EPA 351 mg/d (−8.9 mmHg, 95% CI: 14.9 to – 2.9), inorganic nitrate (−7 mmHg, 95% CI: 12.9 to −1.1), DHA 660 mg/d + EPA 330 mg/d (−1.3 mmHg, 95% CI: 1.4 to −1.2), tart cherry juice (−9.5 mmHg, 95% CI: 12.2 to −6.8), vitamin D 2000 IU/d (−1.3 mmHg, 95% CI: 1.4 to −1.2) reduced sBP in a statistically significant manner, while supplementation with protein resulted in an increase in sBP in older adults (1.9 mmHg, 95% CI: 1.4 to 2.4).

#### 3.4.2 Pairwise comparison of diet supplements on diastolic blood pressure in older adults

Fig. 4B shows the results of the effects of the diet supplements on dBP. Compared with placebo, supplementation with DHA 660 mg/d + EPA 330 mg/d (−0.7 mmHg, 95% CI: 0.7; −0.7), protein (−0.9 mmHg, 95% CI: 1.2; −0.6), 300 mg/d of resveratrol (−5.06 mmHg, 95% CI: 7.6; −2.6), 2000 IU/d of vitamin D (−0.1 mmHg, 95% CI: 0.1; −0.1) reduced dBP in a statistically significant manner, while inorganic nitrate resulted in an increase in dBP in older adults (5 mmHg, 95% CI: 1.1; 8.9).

#### 3.4.3 Pairwise comparison of diet supplements on mean blood pressure in older adults

Fig. 4C shows the results of the effects of the diet supplements on mBP. Compared with placebo, supplementation with DHA 660 mg/d + EPA 330 mg/d (−0.45 mmHg, 95% CI: 0.5 to −0.5), protein (−2.96 mmHg, 95% CI: 3.3 to −2.6), 2000 IU/d of vitamin D (−0.05 mmHg, 95% CI: 0.1 to −0.1) reduced mBP in a statistically significant manner, while supplementation with inorganic nitrate and tart cherry juice resulted in an increase in mean blood pressure in older adults (4 mmHg, 95% CI: 0.1 to 7.9 and 3.2 mmHg, 95% CI: 0.5 to 5.9, respectively).

### 3.5 Rank probabilities

Fig. S2 shows the detailed results of the ranking. The ranking of the effectiveness of each intervention for different outcomes showed that tart cherry juice supplementation was ranked as superior in comparison to other supplementations for reduction in sBP (Fig. S2a, Table S1), and resveratrol 300 mg/day in dBP (Fig. S2b, Table S2) and protein supplementation in mBP (Fig. S2c, Table S3). In terms of sBP reduction tart cherry juice supplementation was more effective than: 2000 IU/day of vitamin D (−8.20 [CI: 10.86; −5.54]), 4000 IU/day of vitamin D (−7.47 [−11.08; −3.87]), 400 IU/d of vitamin E (−2.70 [−11.08; 5.68]), 100mcg/d of vitamin K2 (−13.50 [−19.40; −7.60]) (Table S4). However, tart cherry juice supplementation was less effective than the pooled effect of placebo (9.50 [6.84; 12.16]). In terms of dBP reduction supplementation with 300 mg/d of resveratrol was more effective than tart cherry juice (−5.16 [−15.07; 4.75], 2000 IU/day of vitamin D (−4.96 [−7.47; −2.45], 4000 IU/day of vitamin D (−6.00 [−8.93; −3.07]), 400 IU/d of vitamin E (−6.16 [−11.54; −0.78]), 100 mcg/d of vitamin K2 (−3.06 [−7.80; 1.68]) (Table S5). However, 300 mg/d of resveratrol was less effective than 1000 mg/d of resveratrol (1.40 [−3.91; 6.71]), which was the second most effective supplementation in dBP reduction according to the P score. In addition, resveratrol supplementation was less effective than the pooled effect of placebo in dBP reduction (5.06 [2.55; 7.57]). In terms of mBP reduction supplementation with protein was more effective than 1000 mg/d of resveratrol (−0.76 [−7.13; 5.61], tart cherry juice (−6.16 [−8.88; −3.44]), 2000 IU/day of vitamin D (−2.91 [−3.27; −2.54]), 4000 IU/day of vitamin D −4.06 [−5.91; −2.21]), 400 IU/d of vitamin E (−5.94 [−11.76; −0.12]), 100mcg/d of vitamin K2 (−0.30 [−3.92; 3.33]) (Table S6). However, protein supplementation was less effective than 300 mg/d of resveratrol (0.24 [−3.96; 4.45]) and placebo (2.96 [2.60; 3.32]) in mBP reduction (Table S6). As Tables S4–S6 and Fig. S2 present, none of the examined forms of supplementation were significantly different from each other in blood pressure reduction.

### 3.6 Meta-regression analysis

Both baseline blood pressure and supplementation duration were examined as moderators of treatment effect in comparison to placebo for all dietary supplements pooled together. No statistically significant effect of baseline blood pressure and supplementation duration were found in moderation of response to supplementation in sBP (estimate = 0.04, p = 0.81 for sBP at the baseline and estimate = 0.02, p = 0.18 for supplementation duration in weeks) dBP (estimate = 0.20, p = 0.25 for dBP at the baseline and estimate<0.001, p = 0.64 for supplementation duration in weeks) and mBP (estimate = 0.11, p = 0.52 for mBP at the baseline and estimate = −0.01, p = 0.16 for supplementation duration in weeks, respectively).

### 3.7 Comparison-adjusted funnel plot

Fig. S3 shows a comparison-adjusted funnel plot for the model for effects on sBP (Fig. S3a), dBP (Fig. S3b), mBP (Fig. S3c). All studies on the funnel plot were symmetrically distributed on either side of the vertical line of X = 0, indicating that there was no significant publication bias. P-values from the linear regression test of funnel plot asymmetry (Egger method) was 0.32, indicating no evidence for publication bias.

## 4 Discussion

In terms of sBP reduction tart cherry juice supplementation was more effective than: 2000 IU/day of vitamin D, 4000 IU/day of vitamin D, 400 IU/d of vitamin E, 100mcg/d of vitamin K2. Nevertheless, tart cherry juice supplementation was less effective than the pooled effect of placebo. In terms of dBP reduction supplementation with 300 mg/d of resveratrol was more effective than: tart cherry juice, 2000 IU/day of vitamin D, 4000 IU/day of vitamin D, 400 IU/d of vitamin E, 100 mcg/d of vitamin K2. Nevertheless, 300 mg/d of resveratrol was less effective than 1000 mg/d of resveratrol, which was the second most effective supplementation in dBP reduction according to the P score. In addition, resveratrol supplementation was less effective than the pooled effect of placebo in dBP reduction. In terms of mBP reduction supplementation with protein was more effective than: 1000 mg/d of resveratrol, tart cherry juice, 2000 IU/day of vitamin D, 4000 IU/day of vitamin D, 400 IU/d of vitamin E, 100mcg/d of vitamin K2. However, protein supplementation was less effective than 300 mg/d of resveratrol and placebo in mBP reduction.

### 4.1 Potential physiological mechanism underlying effects of diet supplements on blood pressure in older adults

None of the examined forms of supplementation were significantly different from each other in blood pressure reduction. Supplementation with 480 ml per day with tart cherry juice has been shown to reduce sBP by a mean of −9.5 compared to a placebo in the study by Chai et al. and has been assessed as more effective in sBP reduction than other supplements, averaged overall competing supplements in sBP reduction based on the P score [29]. It has been shown that both tart cherry and some of its metabolites could influence inflammatory signalling pathways [35]. It has been demonstrated that tart cherry juice may be cardioprotective in older adults not only because of its anti-inflammatory properties but also anti-oxidative effects [36]. The consumption of tart cherry juice significantly reduced the levels of inflammatory biomarker C-reactive protein and reduced oxidative stress by increasing the DNA repair activity of OGG1 in older adults [36]. Montmorency tart cherries are rich in flavonoids such as isorhamnetin, kaempferol, quercetin, catechin, epicatechin, procyanidins, and anthocyanins, that act as strong antioxidants [37]. One of the anthocyanins found in tart cherries, cyanidin-3-glucoside, increases the expression of endothelial nitric oxide (NO) synthase (eNOS) that produces NO, an important vasodilatator [38]. Also, the prolonged consumption of cherries decreases the plasma concentration of endothelin-1 (ET-1) which is a vasoconstrictor [38]. These effects may eventually contribute to the reduction of blood pressure [38]. The blood pressure-lowering effect of tart cherry juice may be also explained by its potassium content [39]. In addition, results of a meta-analysis of randomized controlled trials potassium supplementation reduces BP in a modest, but significant manner [[40], [41]]. Dietary potassium intake appears to cause natriuresis and prevent retention of sodium and thus lower BP [[42], [43]]. In turn, it seems that reduction of dietary potassium influences on decrease in serum potassium level in normokalaemia, and is linked to a diminished mortality in patients with CKD [[44], [45]].

Supplementation with 300 mg per day of resveratrol has been shown to reduce diastolic and mean blood pressure by a mean of −5.06 and −3.2 mmHg, respectively, compared to a placebo in the study by Anton et al. and has been assessed as a more effective in dBP reduction than another supplements, averaged over all competing supplements in dBP reduction based on the P score [24]. Resveratrol seems to modulate a wide range of cell signaling molecules; including enzymes, cytokines, kinases, transcription factors, and other molecules, described thoroughly elsewhere [46]. The blood pressure-lowering effect of resveratrol may be explained by its ability to increase the expression of eNOS, which promote the production of NO in endothelial cells, and inhibits the production of vasoconstrictor ET-1 [47]. In addition, resveratrol reduces oxidative stress in endothelial and smooth muscle cells, vascular inflammation and ameliorates vascular remodelling and arterial stiffness, which is important to maintaining normal blood pressure [47]. Moreover, resveratrol induces a calorie restriction-like effect by reducing the sleeping and resting metabolic rate, which is beneficial in controlling blood pressure [48]. The mechanism by which resveratrol could also reduce blood pressure, is the increase in sirtuins expression, specifically sirtuin (silent mating type information regulation 2 homolog) 1 (SIRT1), which ameliorates oxidative stress and is considered to be an anti-aging molecule [48]. Resveratrol has a very high tolerability profile in humans [49]. However, further studies on the potential side effects of resveratrol and administration methods that would lead to its higher bioavailability are needed [50].

A somewhat surprising finding might be the fact that lower dosages of resveratrol 300 mg per day seemed to be more effective than the higher tested dose (1000 mg/d) in dBP reduction. The same with comparing the effectiveness of 2000 IU/day to 40,000 IU/day of vitamin D: the former seemed to be more effective in dBP reduction. It has been shown that a dosage of resveratrol of 1000 mg/day or higher might inhibit cytochrome P450 isoenzymes such as CYP3A4, CYP2C9, and CYP2D6 while stimulating CYP1A2, which in turn could interfere with the action of many other drugs [51]. Therefore Chudzińska et al. and Shaito et al. suggest that further studies should be conducted due to the lack of sufficient data on resveratrol effects and potential adverse effects of different dosing protocols [50,52]. In the case of vitamin D, the effect might be explained based on the fact that two studies incorporated supplementation of 2000 IU/day, while one study examined the effect of 4000 IU/day. Therefore, a group with a lower dose had a relatively higher statistical power due to the higher sample size. In summary, in the recent meta-analysis supplementation with vitamin D was shown to be not effective in BP reduction in the general population [53]. In contrary, it was observed that vitamin D supplementation might effective in lowering BP in older adults with vitamin D deficiency and elevated BP [54]. Exact mechanism of this effect seems to be not yet revealed, however several correlates of change in response to vitamin D supplementation have been found, including parathyroid hormone, serum calcium, renin, and angiotensin II concentrations [54]. It has been suggested that an increased risk of hypertension might apply to patients with decreased levels of vitamin D metabolite level (25(OH)D). However, further studies are needed to describe the relationship between those two factors, which seems to be non-linear in nature [55]. Meta-analysis looking at the effects of Vitamin D supplementation did not have enough evidence to prove its effectiveness on indicators that are risk factors for cardiovascular disease [56]. Al Mheid and Quyyumi suggested that the relationship between vitamin D deficiency and cardiovascular disease might be a spurious relationship, where an unknown third variable might be related to both and truly explain cardiovascular outcomes [56]. Kord-Varkaneh et al. suggested that vitamin D dosages higher than 1000 IU per day and supplementation longer than 12 weeks might lead to a substantial insulin-like growth factor 1 (IGF-1) level increase [57]. Nevertheless, more studies should aim to examine the dose-dependent health benefits and potential adverse effects of vitamin D supplementation in older adults. A study by Sadat-Ali et al. using a general sample, indicated that the dose of 2000 IU of Vitamin D is too low to keep a level of 25(OH)D above 30 ng/ml [58].

Vitamin D may affect blood pressure by having an influence on endothelial cells and smooth muscle cells. In addition, the role of vitamin D deficiency in renin-angiotensin-aldosterone system activation, abnormal nitric oxide regulation, oxidative stress, and dysregulation of inflammatory pathways have been proposed [55]. Vitamin D deficiency might be related to vascular dysfunction, increased stiffening of arteries, hypertrophy of the left ventricle, and worsened indicators of diabetes, hyperlipidaemia and hypertension, and higher cardiovascular mortality [56].

In the current network meta-analysis, two studies have implemented supplementation with omega-3 [25,31]. Bischoff-Ferrari et al. [25] have used 330 mg of EPA plus 660 mg of DHA from marine algae, while Mengelberg et al. have used 1491 mg of triglyceride DHA and 351 mg of EPA [31]. In addition, Mengelberg et al. have provided information on the quantity of vitamin E as well as an anti-oxidant and orange oil (10 mg per capsule) to help mask the taste of the oils. Because of differences in dose and potential qualitative differences, that two interventions were not combined in the current study. Omega-3 components inhibit the production of pro-inflammatory eicosanoids and produce anti-inflammatory lipid mediators, namely resolvins and protectins [55]. Bernasconi et al. concluded that omega-3 might prevent cardiovascular disorders in a dose-dependent manner [58]. However, caution should be taken in drawing such a conclusion, as none of the analysed studies applied a dose higher than 6 g/d, therefore those results cannot be extrapolated above this dosage [58]. Further studies on omega-3 dose-response effects are needed in older populations, as most anti-inflammatory nutrients and drugs, could have detrimental health effects when dosage is excessive [58]. Results of the meta-analysis showed that omega-3 supplementation might diminish cardiovascular mortality in patients with chronic kidney disease (CKD) on hemodialysis [59].

In pairwise comparison with placebo protein supplementation reduced dBP and therefore mBP, while it increased sBP, all in a statistically significant manner. In our meta-analysis adding 30 g/d of protein led to an increase in sBP and a reduction of dBP, which in turn led to a reduction in mBP. Reduction of body fat mass might be a potentially confounding factor, as it might facilitate blood pressure reduction [60]. What seems important, is changes in body composition, which has been not reported by Hodgson et al. [26]. Therefore, it is uncertain if observed effects on blood pressure could be attributed to changes in body composition.

Compared with placebo, supplementation with inorganic nitrate reduced sBP in a statistically significant manner while inorganic nitrate resulted in an increase in dBP and therefore in mBP in older adults in the current study. Those results were obtained despite a relatively short period of the supplementation period, which was 4 weeks [28]. It has been suggested that inorganic nitrate might act on changing the renin-angiotensin-aldosterone system, arginase, and eNOS, as well as indicate anti-inflammatory and antioxidative effects [61]. Another potential explanation of the observed results in the case of inorganic nitrate effects is the fact that the sample size was relatively small (n = 13) and therefore those results need to be replicated on a bigger sample size before drawing definite conclusions.

The so-called “inflammaging” has been described to play a role related to pathomechanism of disorders that are characterized by a higher prevalence in older adults [61]. Interaction of the effects of inflammation, oxidative stress, and endothelial dysfunction presumably influence on hypertension development in older adults [3]. In summary, what might be a potential common denominator of the physiological mechanism underlying the effects of some diet supplements on blood pressure reduction in older adults is the reduction in the level of chronic inflammation and restoration of oxidative-antioxidative balance.

### 4.2 Limitations of the above study and future directions

Firstly, the risk of bias was assessed as low in five of the twelve analysed studies. Some concerns apply to three studies and four studies were burdened with high risk. Therefore, the overall quality of the analysed studies might be assessed as low. Data on the baseline, final, and change of the main outcome were underreported in all of the analysed studies except one [25].

Secondly, the network itself is not free from limitations and should be regarded as rather sparse in terms of the amount of information it contains, what in turn might lead to potential low precision of obtained results [62]. On the other hand, the network in the current study was composed of twelve studies including 2736 participants in the intervention group and 2631 in placebo. The number of analysed studies is rather low and only one intervention (i.e. supplementation with 2000 IU/day of vitamin D) was applied in two studies. The small number of available trials for direct comparisons as well as for the different types of dietary supplements may have influenced our findings. Since for most supplementations, the number of studies was only 1, the effect of supplementation duration analysed in meta-regression was confounded with supplementation type.

Thirdly, the analysed study protocols and samples at baseline were not homogenous. However, the results of the meta-regression showed no statistically significant relationship between baseline blood pressure and supplementation duration with the blood pressure changes (p = 0.18, p = 0.64, and p = 0.16 for sBP, dBP, and mBP, respectively). Additionally, the reduction of body fat mass might potentially be a confounding factor, as it might facilitate blood pressure reduction [60]. For this reason, body composition should be controlled in further intervention-based studies, where reduction of body fat should be assessed as a potential indirect effect of dietary supplementations, which in turn could translate into blood pressure reduction. Lastly, we cannot determine the impact energy balance changes and related behaviours, such as physical activity, the timing of meals, their quantity and quality in terms of macro- and micronutrients had on blood pressure.

Fourthly, effects of particular diet supplements should be accompanied by data on influence on other parameters, including those that could directly be connected to essential HTN pathology, as renal and vascular mechanisms, heart rate (HR), stroke volume, total peripheral resistance (TPR) or indicators of the renin–angiotensin system activity [63]. In addition,stress hormones, and cholesterol levels should be assessed, to better understand mechanism of action of a specific dietary supplement. McMahon et al. [23] provided no information on effects of vitamins B on HR and cholesterol of participants. However, Stanhewicz and Schneider provides data on cholesterol and its fractions (high-density lipoproteins (HDL) and low-density lipoproteins (LDL) just for baseline [28,32]. Jessup et al. provide information on accompanying changes in VO2max and body mass in response to vitamin E supplementation [33]. Hodgson et al. [26] reported changes of body mass in response to protein supplementation. Bischoff-Ferrari et al. examined effects on six different health-related outcomes, including physical performance, infection rate, and cognitive function [2425 In contrast, Anton et al. [24] reported effects of resveratrol supplementation on multiple parameters aside of BP, including glucose level, and liver enzymes, however no information on HR and cholesterol was provided. Tomson and Jaime reported no significant effects of diet supplements on HR [22,30]. Fulton et al. [27] observed no significant effects were seen on carotid intima-media thickness, cholesterol, B-type natriuretic peptide (BNP) or C-reactive protein (CRP) levels. Chai et al. [29] noted no effects of tart cherry juice on insulin resistance index, total cholesterol, HDL. Participants in the tart cherry group had lower levels of LDL cholesterol [29]. Mengelberg et al. [31] found significant effects of DHA supplementation on lowering depression and anxiety intensity.

Fifthly, further studies on the effects of diet supplements on BP should consider also the mediating role of comorbidities present at baseline, such as diabetes type II. It would require possibly larger sample sizes than in the most of analysed studies in the current meta-analysis. For instance, in patients with type 2 diabetes, the hypotensive effects of sodium glucose cotransporter 2 inhibitors and glucagon-like peptide 1 receptor agonists were significantly associated with a reduction in mortality and cardiorenal events [64]. In addition, a hypertensive should be distinguished from a pre-hypertensive state, and the effects of different approaches on BP should be compared. Further potential side effects of further the roles of dietary spermidine for lowering BP [65]. In addition, potential side effects of further treatments should be examined, as, for instance, multiple commonly prescribed drugs might induce orthostatic hypotension [66].

As already has been mentioned, the significance of BP reduction in older patients with baseline SBP ≥160 mmHg to reduce cardiovascular event risk was underlined [13]. In the current meta-analysis, forty-nine studies were excluded because the mean age of samples was not reported or lower than 65 years old. Samples in the included studies with a mean age above 65 could potentially include some participants below 65. Therefore, the applied criterion was not perfect in distinguishing older adults aged 65 years and above. However, no other option was available. Presumably, the effect of aging on blood pressure per se could also potentially be a confounding factor, especially in studies with longer durations. Based on data from longitudinal studies, it is recognized that sBP changes over the lifetime, reaching a peak at 55 years old with a 1.5 mmHg increase per year (95% CI: 1.1, 1.9) in men and 1.4 mmHg per year (95% CI: 1.1, 1.8) in women in this cohort of study [67]. After 65, age-associated sBP increases are attenuated, and in fact, sBP starts to decrease at age 65–70 years old, based on three different longitudinal studies [67]. Therefore, it is advised that future research should implement statistical models that control for the effects age has on participants using dietary supplements to understand their effects on blood pressure, especially in samples where variation in age is high and with a relatively long supplementation program.

## 5 Conclusions

DHA and EPA, inorganic nitrate, omega-3s, tart cherry juice, and vitamin D were more effective in the reduction of sBP and inorganic nitrate, omega-3s, protein, resveratrol, vitamin D supplementation were more effective in the reduction of dBP in a direct comparison to placebo in older adults. None of the examined forms of supplementation were significantly different from each other in blood pressure reduction with a significance level α = 0.05. Caution is needed when interpreting the results of the above study because the overall quality of methodological rigor of analysed studies was assessed as low. Further studies are needed to examine the efficacy of different dosing protocols and combinations of diet supplements in the reduction of blood pressure in older adults.

## Data availability

Data associated with the study has not been deposited into a publicly available repository and data will be made available on request.

## Funding

No funding was received to do this work. SB received support from the 10.13039/100000002National Institutes of Health (T32 DK 007703 and D43 TW010543)

## Competing interests

None of the authors declare any conflicts of interest.

## Ethics approval

Not applicable.

## Human and animal rights and informed consent

This article does not contain any studies with human or animal subjects performed by any of the authors.

## CRediT authorship contribution statement

**Agnieszka Kujawska:** Writing – review & editing, Writing – original draft, Project administration, Methodology, Investigation, Formal analysis, Data curation, Conceptualization. **Sabri Bromage:** Writing – review & editing. **Jose Augusto Simoes:** Writing – review & editing. **Jūratė Zupkauskienė:** Writing – review & editing. **Nicholas McMahon:** Writing – review & editing. **Paweł Zalewski:** Writing – review & editing. **Sławomir Kujawski:** Writing – review & editing, Writing – original draft, Visualization, Validation, Supervision, Software, Project administration, Methodology, Investigation, Funding acquisition, Formal analysis, Data curation, Conceptualization.

## Declaration of competing interest

The authors declare the following financial interests/personal relationships which may be considered as potential competing interests:

Sabri Bromage reports a relationship with 10.13039/100000002National Institutes of Health that includes: funding grants.
