# Supplementary material for: The impact of dietary supplements on blood pressure in older adults: A network meta-analysis of randomized controlled trials
Source: Heliyon. 2024 Feb 8;10(4):e25615. doi: 10.1016/j.heliyon.2024.e25615 (PMC10877265; doi:10.1016/j.heliyon.2024.e25615)
Supplement: Multimedia component 1 [file mmc1.docx]

**Table S1.** P score for supplementation effectiveness in systolic blood pressure changes

| **Diet supplement** | **P score (common)** | **P score (random)** |
| --- | --- | --- |
| tart cherry juice 480 ml/d | 0.9 | 0.9 |
| DHA 1491 mg/d, EPA 351 mg/d | 0.9 | 0.9 |
| NO2- 20 mg/d, NO3- 250 mg/d | 0.8 | 0.8 |
| vitamin E 400 IU/d | 0.8 | 0.8 |
| folic acid 5 mg/d | 0.6 | 0.6 |
| vitamin D 4000 IU/d | 0.6 | 0.6 |
| vitamin D 2000 IU/d | 0.5 | 0.5 |
| DHA 660 mg/d, EPA 330 mg/d | 0.5 | 0.5 |
| placebo | 0.4 | 0.4 |
| resveratrol 300 mg/d | 0.4 | 0.4 |
| resveratrol 1000 mg/d | 0.3 | 0.3 |
| L-citrulline 6 g/d | 0.3 | 0.3 |
| folate 1 mg, vitamin B-12 500 mg, vitamin B-6 10 mg/d | 0.2 | 0.2 |

**Table S2.** P score for supplementation effectiveness in diastolic blood pressure changes

| **Diet supplement** | **P score (common)** | **P score (random)** |
| --- | --- | --- |
| resveratrol 300 mg/d | 0.9 | 0.9 |
| resveratrol 1000 mg/d | 0.8 | 0.8 |
| folate 1 mg, vitamin B-12 500 mg, vitamin B-6 10 mg/d | 0.7 | 0.7 |
| vitamin K2 100mcg/d | 0.7 | 0.7 |
| DHA 1491 mg/d, EPA 351 mg/d | 0.6 | 0.6 |
| DHA 660 mg/d, EPA 330 mg/d | 0.6 | 0.6 |
| protein 30 g/day | 0.6 | 0.6 |
| L-citrulline 6 g/d | 0.5 | 0.5 |
| placebo | 0.4 | 0.4 |
| tart cherry juice 480 ml/d | 0.4 | 0.4 |
| vitamin D 2000 IU/d | 0.4 | 0.4 |
| folic acid 5 mg/d | 0.3 | 0.3 |
| vitamin D 4000 IU/d | 0.3 | 0.3 |

**Table S3.** P score for supplementation effectiveness in mean blood pressure changes

| **Diet supplement** | **P score (common)** | **P score (random)** |
| --- | --- | --- |
| protein 30 g/day | 0.9 | 0.9 |
| resveratrol 300 mg/d | 0.9 | 0.9 |
| vitamin K2 100mcg/d | 0.8 | 0.8 |
| folate 1 mg, vitamin B-12 500 mg, vitamin B-6 10 mg/d | 0.7 | 0.7 |
| resveratrol 1000 mg/d | 0.7 | 0.7 |
| DHA 660 mg/d, EPA 330 mg/d | 0.6 | 0.6 |
| placebo | 0.5 | 0.5 |
| vitamin D 2000 IU/d | 0.5 | 0.5 |
| DHA 1491 mg/d, EPA 351 mg/d | 0.4 | 0.4 |
| L-citrulline 6 g/d | 0.4 | 0.4 |
| vitamin D 4000 IU/d | 0.3 | 0.3 |
| folic acid 5 mg/d | 0.2 | 0.2 |
| tart cherry juice 480 ml/d | 0.2 | 0.2 |

**Table S4.** League table presenting square matrix showing all pairwise comparisons for sBP changes

| DHA 1491 mg/d,EPA 351 mg/d | . | . | . | . | . | -9[-15;-3] | . | . | . | . | . | . | . | . |
| --- | --- | --- | --- | --- | --- | --- | --- | --- | --- | --- | --- | --- | --- | --- |
| -11[-18;-4] | folate 1 mg, vitamin B-12 500 mg, vitamin B-6 10 mg/d | . | . | . | . | 2[-1;5] | . | . | . | . | . | . | . | . |
| -6[-16;4] | 5[-4;13] | folic acid 5 mg/d | . | . | . | -3[-11;5] | . | . | . | . | . | . | . | . |
| -11[-21;-1] | -0[-9;9] | -5[-16;6] | L-citrulline 6 g/d | . | . | 2[-6;10] | . | . | . | . | . | . | . | . |
| -2[-10;6] | 9[2;16] | 4[-6;14] | 9[-1;19] | NO2- 20 mg/d, NO3- 250 mg/d | . | -7[-13;-1] | . | . | . | . | . | . | . | . |
| -8[-14;-2] | 3[0;6] | -2[-10;6] | 3[-5;12] | -6[-12;0] | DHA 660 mg/d,EPA 330 mg/d | -1[-1;-1] | . | . | . | . | 0[0;0] | . | . | . |
| -9[-15;-3] | 2[-1;5] | -3[-11;5] | 2[-6;10] | -7[-13;-1] | -1[-1;-1] | placebo | -2[-2;-1] | -1[-8;7] | -1[-8;7] | 10[7;12] | 1[1;1] | 2[0;5] | 7[-1;15] | -4[-9;1] |
| -11[-17;-5] | -0[-3;3] | -5[-13;3] | 0[-8;8] | -9[-15;-3] | -3[-4;-3] | -2[-2;-1] | protein 30 g/d | . |  | . | . | . | . | . |
| -10[-19;0] | 1[-7;9] | -4[-15;7] | 1[-10;12] | -8[-17;2] | -2[-9;5] | -1[-8;7] | 1[-6;9] | resveratrol 1000 mg/d | 0[-7;8] | . | . | . | . | . |
| -9[-19;0] | 1[-7;10] | -4[-14;7] | 1[-10;13] | -8[-17;2] | -2[-9;6] | -1[-8;7] | 1[-6;9] | 0[-7;8] | resveratrol 300 mg/d | . | . | . | . | . |
| 1[-6;7] | 11[7;16] | 6[-2;15] | 12[3;20] | 2[-4;9] | 8[6;11] | 10[7;12] | 11[9;14] | 10[2;18] | 10[2;18] | tart cherry juice 480 ml/d | . | . | . | . |
| -8[-14;-2] | 3[0;6] | -2[-10;6] | 3[-5;12] | -6[-12;0] | 0[0;0] | 1[1;1] | 3[3;4] | 2[-5;9] | 2[-6;9] | -8[-11;-6] | vitamin D 2000 IU/d | 0[-3;3] |  | . |
| -7[-13;0] | 4[0;8] | -1[-9;7] | 4[-5;13] | -5[-11;1] | 1[-2;3] | 2[0;4] | 4[1;6] | 3[-5;11] | 3[-5;11] | -7[-11;-4] | 1[-2;3] | vitamin D 4000 IU/d |  | . |
| -2[-12;8] | 9[0;17] | 4[-7;15] | 9[-3;20] | -0[-10;10] | 5[-2;13] | 7[-1;15] | 9[1;17] | 8[-3;18] | 7[-4;18] | -3[-11;6] | 5[-2;13] | 5[-4;13] | vitamin E 400 IU/d |  |
| -13[-21;-5] | -2[-8;4] | -7[-16;2] | -2[-12;8] | -11[-19;-3] | -5[-11;0] | -4[-9;1] | -2[-7;3] | -3[-12;6] | -3[-13;6] | -14[-19;-8] | -5[-11;0] | -6[-12;0] | -11[-20;-1] | vitamin K2 100mcg/d |

**Table S5***.* League table presenting square matrix showing all pairwise comparisons for dBP changes

| DHA 1491 mg/d,EPA 351 mg/d | . | . | . | . | . | -1[-6;3] | . | . | . | . | . | . | . | . |
| --- | --- | --- | --- | --- | --- | --- | --- | --- | --- | --- | --- | --- | --- | --- |
| 0[-5;5] | folate 1 mg, vitamin B-12 500 mg, vitamin B-6 10 mg/d | . | . | . | . | -2[-4;0] | . | . | . | . | . | . | . | . |
| -3[-11;4] | -4[-10;3] | folic acid 5 mg/d | . | . | . | 2[-4;8] | . | . | . | . | . | . | . | . |
| -0[-7;6] | -1[-6;5] | 3[-5;11] | L-citrulline 6 g/d | . | . | -1[-6;4] | . | . | . | . | . | . | . | . |
| -6[-13;0] | -7[-11;-2] | -3[-10;4] | -6[-12;0] | NO2- 20 mg/d, NO3- 250 mg/d | . | 5[1;9] | . | . | . | . | . | . | . | . |
| -0[-5;5] | -1[-3;2] | 3[-3;9] | -0[-5;5] | 6[2;10] | DHA 660 mg/d,EPA 330 mg/d | . | . | . | . | . | -1[-1;-1] | . | . | . |
| -1[-6;3] | -2[-4;0] | 2[-4;8] | -1[-6;4] | 5[1;9] | -1[-3;1] | placebo | 1[1;1] | 4[-2;10] | 5[3;8] | -0[-10;9] | 0[-1;2] | -1[-3;1] | -1[-6;4] | 2[-2;6] |
| -1[-5;4] | -1[-3;1] | 3[-3;9] | -0[-5;5] | 6[2;10] | -0[-2;2] | 1[1;1] | protein 30 g/d | . | . | . | . | . | . | . |
| 2[-5;10] | 2[-4;8] | 6[-3;14] | 3[-5;10] | 9[2;16] | 3[-3;9] | 4[-2;10] | 3[-3;9] | resveratrol 1000 mg/d | 1[-4;7] | . | . | . | . | . |
| 4[-2;9] | 3[0;7] | 7[1;13] | 4[-1;9] | 10[5;15] | 4[1;7] | 5[3;8] | 4[2;7] | 1[-4;7] | resveratrol 300 mg/d | . | . | . | . | . |
| -2[-12;9] | -2[-12;8] | 2[-9;13] | -1[-12;10] | 5[-5;15] | -1[-11;9] | -0[-10;9] | -1[-11;9] | -4[-15;7] | -5[-15;5] | tart cherry juice 480 ml/d | . | . | . | . |
| -1[-6;4] | -1[-4;2] | 2[-4;9] | -1[-6;4] | 5[1;10] | -1[-1;-1] | 0[-1;2] | -1[-2;1] | -3[-9;3] | -5[-8;-2] | 0[-9;10] | vitamin D 2000 IU/d | -1[-3;1] | . | . |
| -2[-7;3] | -2[-5;0] | 1[-5;7] | -2[-7;3] | 4[0;8] | -2[-4;0] | -1[-3;1] | -2[-3;0] | -4[-11;2] | -6[-9;-3] | -1[-10;9] | -1[-3;1] | vitamin D 4000 IU/d | . | . |
| -3[-9;4] | -3[-8;2] | 1[-7;8] | -2[-9;5] | 4[-2;10] | -2[-7;3] | -1[-6;4] | -2[-7;3] | -5[-12;3] | -6[-12;-1] | -1[-12;10] | -1[-7;4] | -0[-5;5] | vitamin E 400 IU/d | . |
| 1[-6;7] | 0[-4;5] | 4[-3;11] | 1[-5;7] | 7[1;13] | 1[-3;5] | 2[-2;6] | 1[-3;5] | -2[-9;5] | -3[-8;2] | 2[-8;12] | 2[-3;6] | 3[-2;7] | 3[-3;9] | vitamin K2 100mcg/d |

**Table S6.** League table presenting square matrix showing all pairwise comparisons for mBP changes

| DHA 1491 mg/d,EPA 351 mg/d | . | . | . | . | . | 1 [-4; 6] | . | . | . | . | . | . | . | . |
| --- | --- | --- | --- | --- | --- | --- | --- | --- | --- | --- | --- | --- | --- | --- |
| 3 [-3; 8] | folate 1 mg, vitamin B-12 500 mg, vitamin B-6 10 mg/d | . | . | . | . | -2 [-4; 1] | . | . | . | . | . | . | . | . |
| -2 [-8; 4] | -5 [-9; 0] | folic acid 5 mg/d | . | . | . | 3 [-1; 7] | . | . | . | . | . | . | . | . |
| -0 [-7; 7] | -3 [-8; 3] | 2 [-4; 8] | L-citrulline 6 g/d | . | . | 1 [-4; 6] | . | . | . | . | . | . | . | . |
| -3 [-9; 3] | -6 [-10; -1] | -1 [-7; 5] | -3 [-9; 3] | NO2- 20 mg/d, NO3- 250 mg/d | . | 4 [0; 8] | . | . | . | . | . | . | . | . |
| 1 [-4; 6] | -1 [-4; 1] | 3 [0; 7] | 1 [-4; 6] | 4 [1; 8] | DHA 660 mg/d,EPA 330 mg/d | -0 [0; 0] | . | . | . | . | -0 [0; 0] | . | . | . |
| 1 [-4; 6] | -2 [-4; 1] | 3 [-1; 7] | 1 [-4; 6] | 4 [0; 8] | -0 [0; 0] | placebo | 3 [3; 3] | 2 [-4; 9] | 3 [-1; 7] | -3 [-6; -1] | 0 [0; 0] | -1 [-3; 1] | -3 [-9; 3] | 3 [-1; 6] |
| 4 [-1; 9] | 1 [-1; 4] | 6 [2; 10] | 4 [-1; 9] | 7 [3; 11] | 3 [2; 3] | 3 [3; 3] | protein 30 g/d | . | . | . | . | . | . | . |
| 3 [-5; 11] | 1 [-6; 7] | 5 [-2; 13] | 3 [-5; 11] | 6 [-1; 14] | 2 [-5; 8] | 2 [-4; 9] | -1 [-7; 6] | resveratrol 1000 mg/d | 1 [-4; 6] | . | . | . | . | . |
| 4 [-2; 11] | 2 [-3; 6] | 6 [0; 12] | 4 [-2; 11] | 7 [1; 13] | 3 [-1; 7] | 3 [-1; 7] | 0 [-4; 4] | 1 [-4; 6] | resveratrol 300 mg/d | . | . | . | . | . |
| -2 [-8; 3] | -5 [-9; -1] | -0 [-5; 5] | -2 [-8; 3] | 1 [-4; 6] | -4 [-6; -1] | -3 [-6; -1] | -6 [-9; -3] | -5 [-12; 2] | -6 [-11; -1] | tart cherry juice 480 ml/d | . | . | . | . |
| 1 [-4; 6] | -2 [-4; 1] | 3 [-1; 7] | 1 [-4; 6] | 4 [0; 8] | -0 [0; 0] | 0 [0; 0] | -3 [-3; -3] | -2 [-9; 4] | -3 [-7; 1] | 3 [1; 6] | vitamin D 2000 IU/d | -1 [-3; 1] | . | . |
| -0 [-6; 5] | -3 [-6; 0] | 2 [-2; 6] | -0 [-5; 5] | 3 [-1; 7] | -2 [-3; 0] | -1 [-3; 1] | -4 [-6; -2] | -3 [-10; 3] | -4 [-9; 0] | 2 [-1; 5] | -1 [-3; 1] | vitamin D 4000 IU/d | . | . |
| -2 [-10; 6] | -5 [-11; 2] | 0 [-7; 7] | -2 [-10; 6] | 1 [-6; 8] | -3 [-9; 2] | -3 [-9; 3] | -6 [-12; 0] | -5 [-14; 3] | -6 [-13; 1] | 0 [-6; 7] | -3 [-9; 3] | -2 [-8; 4] | vitamin E 400 IU/d | . |
| 4 [-3; 10] | 1 [-3; 5] | 6 [0; 11] | 4 [-3; 10] | 7 [1; 12] | 2 [-1; 6] | 3 [-1; 6] | -0 [-4; 3] | 0 [-7; 8] | -1 [-6; 5] | 6 [1; 10] | 3 [-1; 6] | 4 [0; 8] | 6 [-1; 12] | vitamin K2 100mcg/d |

*
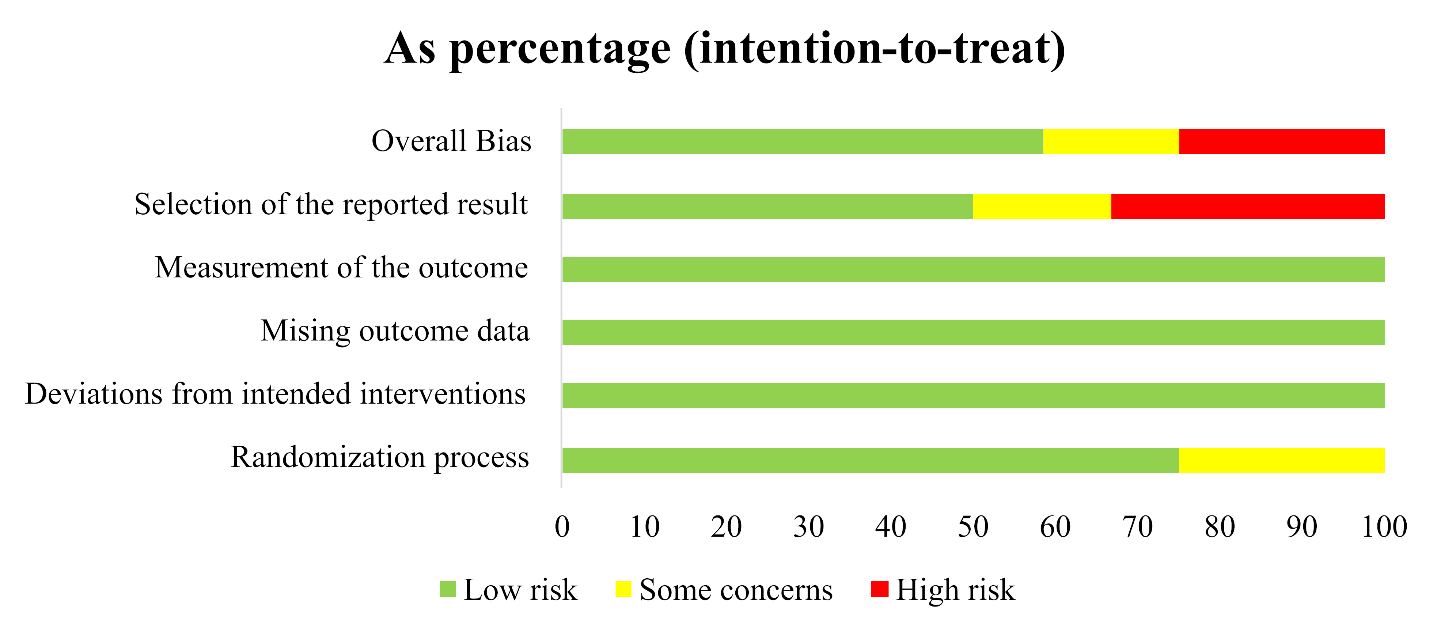
*

**Figure S1.** The overall result of Risk of Bias analysis. *The width of the red bar corresponds to the percentage of high risk of bias in analysed studies, the width of the yellow bar corresponds to the percentage of studies with some concerns related to bias, and the width of the green bar indicates the percentage of studies with low risk of bias.*

**
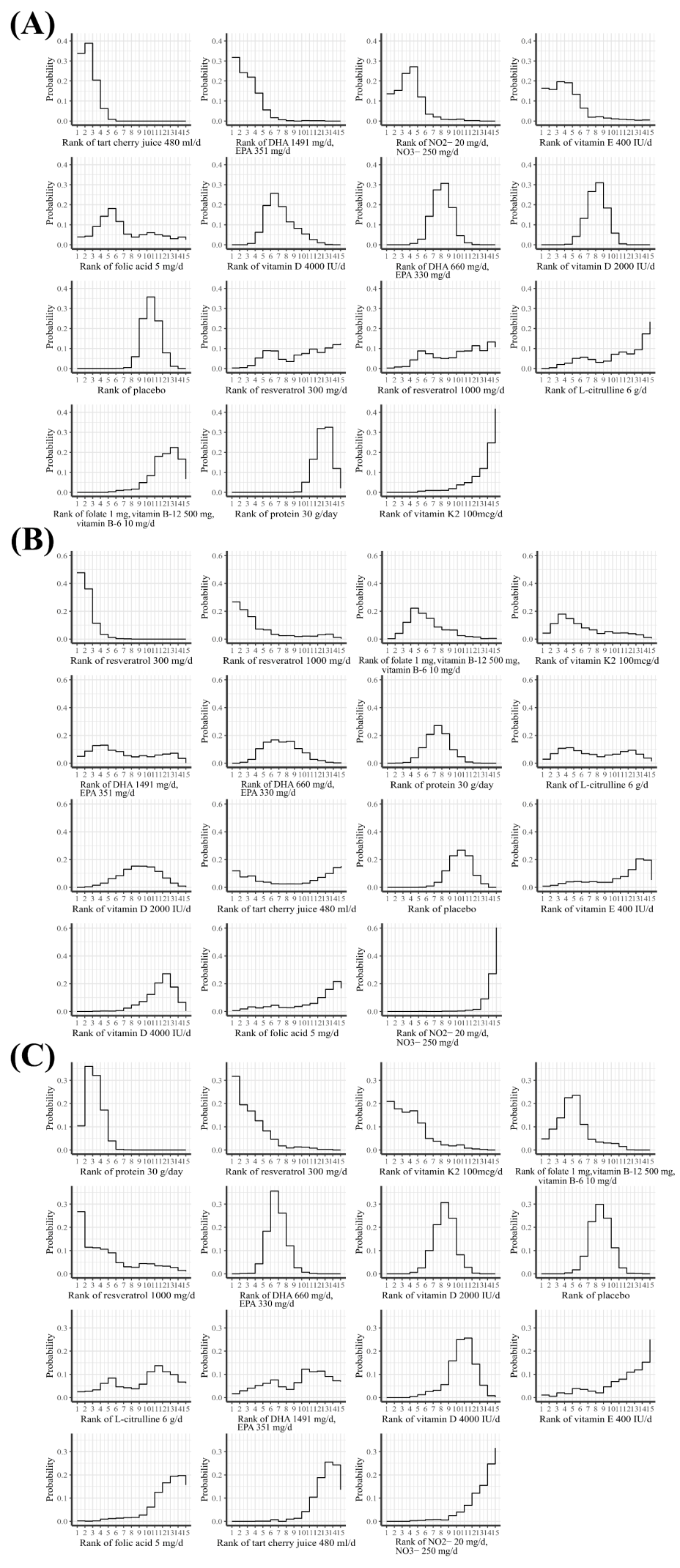
**

**Figure S2.** Rank probabilities for the superiority of supplementation methods. *Panel a) shows effects on systolic blood pressure, b) on diastolic blood pressure, c) on mean blood pressure*

**
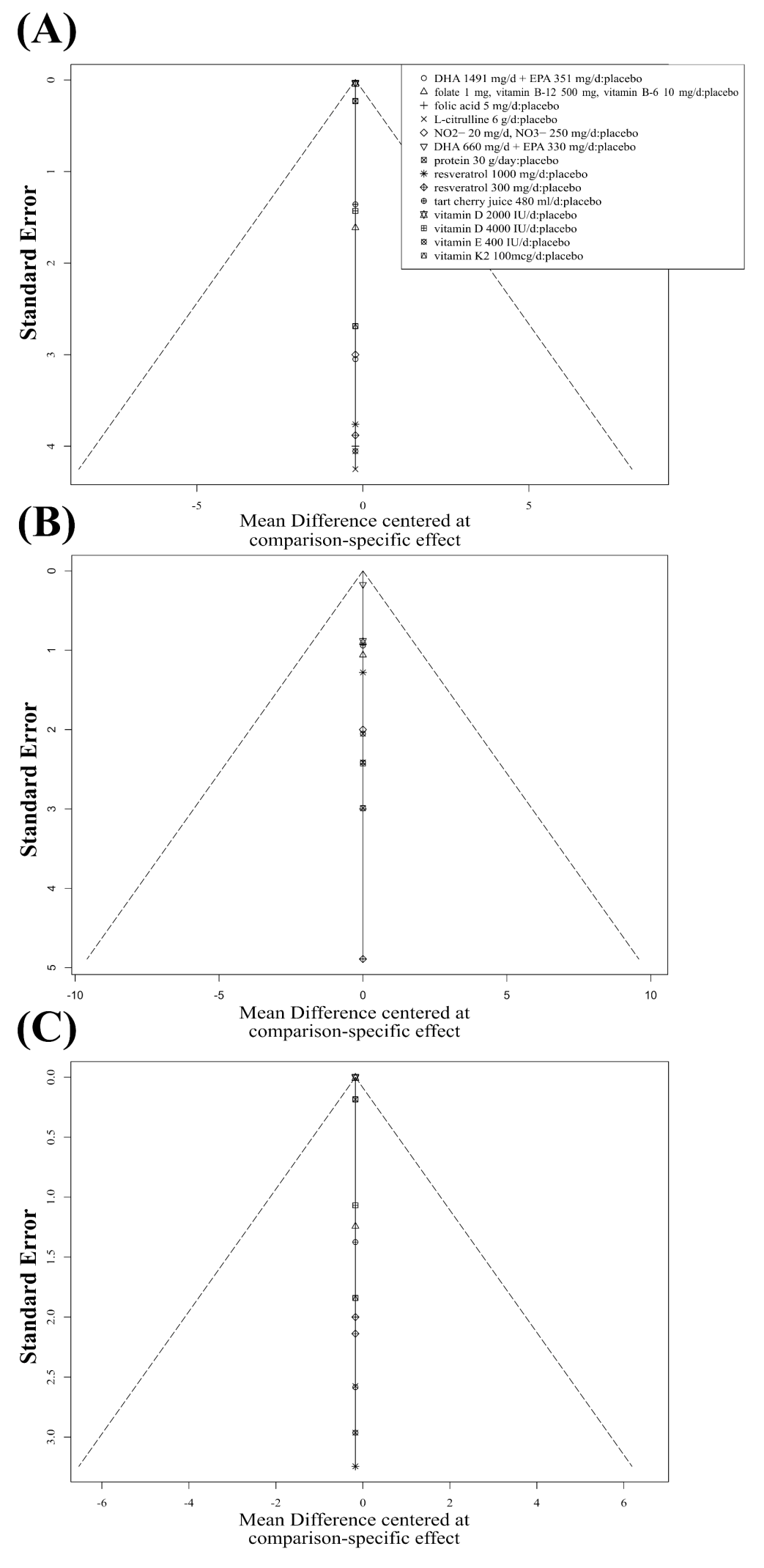
**

**Figure S3.** Comparison-Adjusted Funnel Plot. *Models assessing supplementation effects on a) systolic blood pressure, b) diastolic blood pressure, c) mean blood pressure*
